# Clinical features, treatment and outcome in neurosarcoidosis: systematic review and meta-analysis

**DOI:** 10.1186/s12883-016-0741-x

**Published:** 2016-11-15

**Authors:** Daan Fritz, Diederik van de Beek, Matthijs C. Brouwer

**Affiliations:** Department of Neurology, Academic Medical Centre, Amsterdam Neuroscience, Meibergdreef 9, Amsterdam, 1105AZ The Netherlands

**Keywords:** Neurosarcoidosis, Clinical neurology, Auto-immune disease, Systemic disease, Systematic review, Meta-analysis

## Abstract

**Background:**

Neurosarcoidosis is a rare variant of sarcoidosis and is only described in small cohort studies. We define clinical features, treatment and outcome of patients with neurosarcoidosis over the last 35 years.

**Methods:**

We performed a systematic review and meta-analysis of studies on neurosarcoidosis published between 1980 and 2016. Studies were included if they reported at least 5 cases. Studies describing one specific neurological presentation were excluded.

**Results:**

We identified 29 articles describing 1088 patients diagnosed between 1965 and 2015. Neurosarcoidosis occurred in 5% of patients with systemic sarcoidosis. Mean age at presentation was 43 years and neurological symptoms were the first clinical manifestation of sarcoidosis in 52%. The most commonly reported feature of neurosarcoidosis was cranial neuropathy in 55%, with the facial and optic nerve most commonly affected, followed by headache in 32%. Pleiocytosis and elevated CSF protein were found in 58 and 63%. MRI of the brain showed abnormalities in 70%. Chest X-ray, chest CT, or gallium-67-scintigraphy showed findings consistent with sarcoidosis in 60%, 70% and 69%, respectively. First line therapy with corticosteroids was initiated in 434 of 539 patients (81%). Second and third line therapy was started in 27 and 9%. Outcome consisted of complete remission in 27%, incomplete remission in 32%, stable disease in 24%, deterioration in 6% and death in 5%.

**Conclusion:**

Neurosarcoidosis has a heterogeneous clinical presentation and the diagnosis can be difficult because of low sensitivity of ancillary investigations. New treatments have emerged, but nevertheless one third of patients do not respond to treatment. Prospective cohort studies and RCTs on treatment are urgently needed.

**Electronic supplementary material:**

The online version of this article (doi:10.1186/s12883-016-0741-x) contains supplementary material, which is available to authorized users.

## Background

Sarcoidosis is a multisystem granulomatous inflammatory disease of unknown aetiology, that typically affects young adults [1]. The incidence varies throughout the world, but is estimated to be between 10 and 20 per 100.000 population [2]. It mainly affects lung, skin and eyes, and has been reported to involve the nervous system in 5–20% [3, 4]. Neurologic manifestations described are cranial nerve palsy, aseptic meningitis, peripheral neuropathy and myopathy [5]. Data on neurosarcoidosis are mostly derived from single-centre retrospective studies and vary considerably between studies [6]. Treatment is based on expert opinion, and no randomized controlled trials have been done comparing treatment in patients with neurosarcoidosis [7].

Diagnostic criteria for neurosarcoidosis have been proposed based on a clinical presentation suggestive of neurosarcoidosis, results of ancillary investigation and exclusion of other diagnoses [5, 8–10]. A definite diagnosis of neurosarcoidosis is met in only a minority of patients because this needs histologic confirmation of non-caseating granulomas of affected nervous system tissue. A probable diagnosis is defined as evidence of nervous system inflammation on magnetic resonance imaging (MRI) or cerebrospinal fluid (CSF; elevated protein, cells, immunoglobin G indices, or presence of oligoclonal bands) in combination with evidence of systemic sarcoidosis with histological confirmation and/or at least two of the indirect indicators consisting of fluorodeoxyglucose positron emission tomography (FDG-PET), gallium scan, chest imaging, and serum angiotensin-converting enzyme. Possible neurosarcoidosis is defined a clinical suspicion and exclusion of other diagnoses, but above mentioned criteria are not met.

We performed a systematic review and meta-analysis to determine clinical features, treatment, and outcome of patients with neurosarcoidosis over the last 35 years.

## Methods

PubMed and Embase were searched using the search terms “neurosarcoidosis”, “sarcoidosis” and “nervous system”. Studies written in Dutch, English, French, German, or Spanish published between 1980 and March 2016 were considered for inclusion. Studies were included in the meta-analysis if they reported at least 5 cases of neurosarcoidosis, involving at least intracranial manifestations other than isolated hypothalamo-pituitary neurosarcoidosis. Studies were excluded if they: 1) reported only on a specific subset of neurosarcoidosis manifestations (e.g., neuro-ophthalmic or spinal cord neurosarcoidosis); or 2) were duplicate publications. Clinical features described in at least five case studies are reported. Data on study characteristics, demographic features, clinical manifestations, ancillary investigations, treatment, and outcome were systematically scored by DF and MB. Therapy was classified as first line, second line, or third line therapy. First line therapy consists of corticosteroid treatment, second line treatment consists of immunosuppressive therapy with methotrexate, azathioprine, mycophenolate mofetil, cyclosporine A, or (hydroxyl) chloroquine, and third line therapy either consists of cyclophosphamide or immunomodulatory medication (tumor necrosis factor-alpha inhibitors (TNF-alpha) or B-cell targeted therapy) [3]. Remission was defined as complete improvement, without residual symptoms. Favourable outcome was defined as remission, either complete or incomplete, and no need for alternative immunosuppressants.

We performed a pooled reanalysis of all published data in the included studies. Because of heterogeneity between studies, all data is presented as a number for which a certain characteristic is present out of the total number of patients for which it was described (n/N [%]) and a 95% confidence interval (95% CI) was used. For continuous data the standard deviation (SD) was used. Heterogeneity between studies was calculated for all reported variables using the Cochrane Q and I^2^ statistical tests.

## Results

**Fig. 1:**
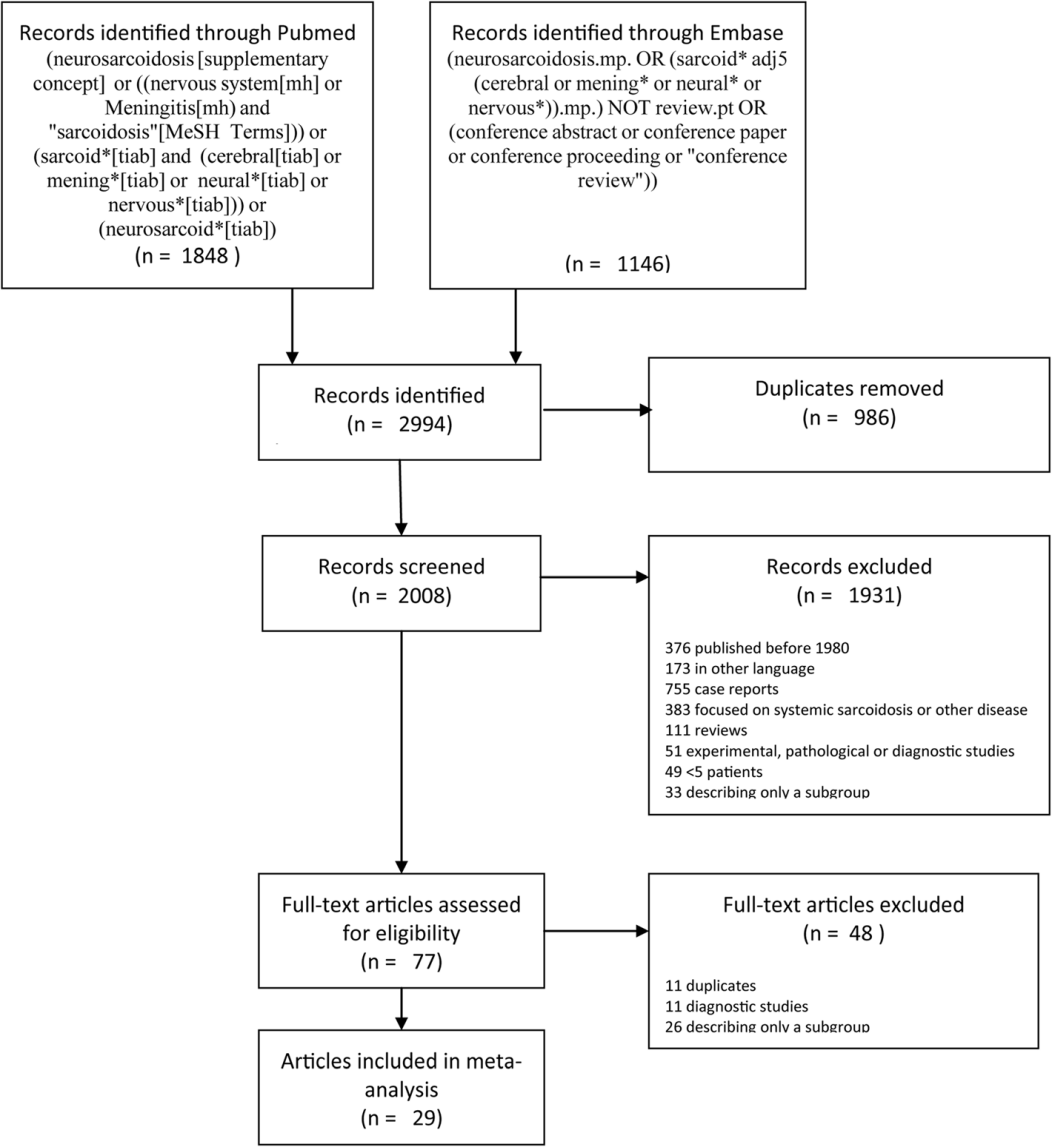
Flow chart of inclusion of studies

Our search identified 2994 articles of which 29 studies remained after screening (Fig. 1), including 1088 patients diagnosed from 1965 to 2015 [6, 8, 9, 11–36]. The number of patients per study ranged from 5 to 305 patients (median 27, interquartile range [IQR] 13–37, Additional file 1: Table S1). The individual study periods varied between 3 and 31 years (median 13 years, IQR 9–15). Only one of 29 studies was performed prospectively (3%); [6] 24 of 29 (83%) were single-centre studies [6, 9, 11–19, 21–27, 29–31, 34–36]. Inclusion criteria were described in 27 of 29 studies (93%) and were based on the Zajicek criteria in 11 studies [8, 9, 25, 27–31, 33, 35, 36]. Four studies included patients with definite or probable neurosarcoidosis according to these criteria, and seven included definite, probable, or possible neurosarcoidosis [8, 9, 25, 27–31, 33, 35, 36]. Eleven studies included biopsy-proven sarcoidosis [6, 11, 14, 16, 18, 20–23, 32, 34]. Five studies included patients with the diagnosis based on either histological evidence of non-caseating granulomas or clinical and radiologic findings consistent with the diagnosis sarcoidosis [13, 15, 17, 19, 24]. Twelve studies considered only central nervous system involvement of neurosarcoidosis [8, 9, 12, 17, 18, 24, 26–28, 30, 33, 34]. Calculation of the heterogeneity of data reported in the pooled study results showed significant heterogeneity (*I* ^2^ > 50%) occurred for 48 of 110 studied parameters (Additional file 1: Table S2 A-C).

### Patient characteristics

Neurosarcoidosis was diagnosed in 246 cases of 5263 reported cases of systemic sarcoidosis (5%, range 2–26%) [6, 13–15, 19, 22, 31]. Mean age at presentation was 43 years (range 11–84 years, SD 11.57, recalculated from 27 studies including 967 patients; Table 1). Overall, 602 of 1088 patients (55%, 95% CI 52–58%) were female. Ethnicity was reported in 739 patients of which 461 patients (62%) were Caucasian and 218 patients (29%) were of African descent. In total 652 patients were diagnosed according to Zajicek diagnostic criteria: [8, 9] 159 patients (25%) were classified as having definite neurosarcoidosis, 371 (59%) as probable neurosarcoidosis and 95 (15%) as possible neurosarcoidosis.

**Table 1:** Patient Characteristics

Characteristic	n/N^a^ (%)	Characteristic	n/N^a^ (%)
Age, yr., mean^b^(SD)	43.1 (11.57)	Neurological symptoms	
Sex, male	486/1088 (45)	Headache	271/860 (32)
Ethnicity		Sensory abnormalities	138/484 (29)
Caucasian	461/739 (62)	Hypaesthesia	39/174 (22)
African	218/739 (29)	Paraesthesia	69/334 (21)
Other	27/739 (4)	Neuropathic pain	25/216 (12)
Unknown	33/739 (4)	Gait abnormalities	128/466 (28)
Presentation of disease		Visual impairment	105/450 (23)
History of sarcoidosis	229/728 (31)	Fatigue	47/202 (23)
Systemic sarcoidosis at admission	256/440 (58)	Motor abnormalities	103/552 (19)
Primary neurological presentation	344/662 (52)	Hemiparesis	41/454 (9)
Isolated neurosarcoidosis	169/770 (22)	Paraparesis	37/348 (11)
Systemic involvement		Ataxia	64/387 (17)
Pulmonary	316/469 (67)	Vertigo	39/277 (14)
Eye	117/469 (25)	Hearing impairment	97/716 (14)
Dermatological	99/469 (21)	Seizures	132/965 (14)
Rheumatological	99/469 (21)	Nausea	11/84 (13)
Otorhinolaryngeal	43/469 (9)	Diplopia	41/361 (11)
Hepatic	37/469 (8)	Micturition abnormalities	23/206 (11)
Cardial	26/469 (6)	Dysarthria	36/413 (9)
Constitutional symptoms	106/469 (23)	Dysphagia	44/481 (9)
Cranial nerve palsy	572/1047 (55)	Psychiatric symptoms	30/390 (8)
n. VII palsy	227/937 (24)	Nystagmus	11/172 (6)
n. II palsy	190/910 (21)	Papilledema	23/354 (6)
n. V palsy	111/942 (12)	Site of neurological involvement	
n. VIII palsy	102/914 (11)	Spinal cord disease	98/543 (18)
n. VI palsy	63/906 (7)	Peripheral neuropathy	97/558 (17)
n. III palsy	46/869 (5)	Polyneuropathy	56/487 (11)
n. IX–X palsy	32/882 (4)	(Multiple Mononeuropathies)	16/137 (12)
n. I palsy	17/889 (2)	Radiculopathy	7/140 (5)
n. IV palsy	17/869 (2)	Meningitis	105/648 (16)
n. XI palsy	5/882 (1)	Myopathy	43/288 (15)
n. XII palsy	12/882 (1)	Neuro-endocrine^c^	90/1034 (9)
>1 cranial nerve involved	206/724 (28)	Hydrocephalus	52/570 (9)

^a^ n/N: number for which a certain characteristic is present out of the total number of patients for which it was described

^b^ The mean age was recalculated from averages presented in 27 studies (967 patients)

^c^ Panhypopituirism (*n =* 34), Diabetes insipidus (*n =* 21), amenorrhea (*n =* 5), hypothermia/hypotension (*n =* 1), galactorrhoea (*n =* 1), not further specified (*n =* 28)

### Clinical characteristics

Neurological symptoms were the first clinical manifestation of sarcoidosis in 344 of 662 patients (52%; 95% CI 48–56%). Patients diagnosed with neurosarcoidosis had a known diagnosis of sarcoidosis outside the nervous system in 229 of 728 patients (31%, 95% CI 28–35%). Systemic manifestations of sarcoidosis outside the nervous system at any time during disease course occurred in 284 of 339 patients (84%; 95% CI 80–88%), consisting of pulmonary involvement in 338 of 589 patients (57%), ocular sarcoidosis in 117 of 589 patients (20%), lymphadenopathy in 105 of 589 patients (18%), dermatological manifestations in 104 of 589 patients (18%) and rheumatological manifestations in 104 out of 589 patients (18%).

The most commonly reported presenting feature of neurosarcoidosis was cranial neuropathy, which occurred in 572 of 1047 patients (55%, 95% CI 52–58%). Of all cranial nerves, the facial nerve (24%, 95% CI 21–27%) and optic nerve (21%, 95% CI 18–24%) were involved most frequently. Other common presenting features were headache (32%, 95% CI 28–35%), sensory abnormalities (29%, 95% CI 24–33%), and motor symptoms (19%; 95% CI 15–22%) consisting of hemiparesis (9%; 95% CI 6–12%) and paraparesis (11%; 95% CI 7–14%). Meningitis was reported in 105 of 648 patients (16%, 95% CI 13–19%). Spinal cord abnormalities were reported in 18% (95% CI 15–21%) and involvement of the peripheral nervous system in 17% of patients (95% CI 14–21%), mainly consisting of polyneuropathy; myopathy was reported in 43 of 288 evaluated patients (15%; 95% CI 11–9%).

### Ancillary investigations

Results of CSF analysis were described in 22 studies (Table 2); a lumbar puncture was performed in the majority of patients (78%). Pleiocytosis was found in 424 of 730 patients (58%; 95% CI 54–62%), and CSF white cell counts ranged from 5 to 1571 cells per mm^3^. CSF total protein was elevated in 457 of 729 patients (63%, 95% CI 59–66%). The IgG-index was elevated in 36 of 89 evaluated cases (40%, 95% CI 30–51%) with CSF oligoclonal bands present in 77 of 184 patients (46%, 95% CI 35–49%). CSF angiotensin-converting enzyme (ACE) levels were elevated in 189 of 410 patients (46%, 95% CI 41–51%).

**Table 2:** Ancillary investigations on presentation

Characteristic	n/N^a^ (%)	Characteristic	n/N^a^ (%)
Blood chemical tests		Abnormal ancillary investigation	
Serum ACE increased^b^	238/674 (35)	Chest X-ray	277/461 (60)
Serum calcium increased^b^	19/222 (9)	Chest CT	93/132 (70)
ESR >20 mm/h	64/202 (32)	Gallium-67 scintigraphy	81/123 (66)
		Cranial CT	83/168 (49)
Cerebrospinal fluid analysis		Cranial MRI	283/362 (78)
Lumbar puncture performed	774/988 (78)	Parenchymal lesions	191/378 (51)
White cell count (cells/mm^3^)	5–1571	Meningeal enhancement	282/610 (46)
>5 cells/mm^3^	424/730 (58)	Mass lesions	82/501 (16)
Protein (g/L)	0.45–22.4	Cranial nerve enhancement	123/478 (26)
>0.45 g/L^c^	457/729 (63)	Spinal MRI	89/185 (48)
Hypoglycorrhachia^c^	43/312 (14)	Diagnosis	
Increased IgG-index^c^	36/89 (40)	Histopathological confirmation^d^	455/550 (81)
Oligoclonal bands present	77/184 (42)	Definite neurosarcoidosis	159/625 (25)
Increased CSF ACE^c^	189/410 (46)	Probable neurosarcoidosis	371/625 (59)
Normal	90/332 (27)	Possible neurosarcoidosis	95/625 (15)

^a^ n/N: number for which a certain characteristic is present out of the total number of patients for which it was described

^b^ As reported in articles. The normal values of serum calcium were not reported in studies. The normal values of serum ACE varied between > 52 IU/L and >100 IU/L

^c^ As reported in the articles. The normal values of CSF glucose, CSF IgG index and CSF ACE varied between studies (CSF glucose between <35 mg/dL and <0.50 mg/dL, IgG index between >0.6 and >0.7 and CSF ACE between > 1U/L and 3 U/L)

^d^ Total histopathological confirmation, either in the CNS or systemic disease

Cranial MRI showed abnormalities in 283 of 362 evaluated patients (79%, 95% CI 74–83%), consisting of parenchymal lesions in 51% (95% CI 45–56%), and contrast enhancement of the meninges in 46% (95% CI 42–50%) and of cranial nerves in 26% (95% CI 22–30%). Spine MRI showed abnormalities in 89 of 185 evaluated patients (48%; 95% CI 41–55%). Chest X-ray showed findings consistent with pulmonary or lymph node sarcoidosis in 277 of 461 patients (60%, 95% CI 56–65%) and chest CT in 93 of 132 patients (70%, 95% CI 63–78%). Gallium-67-scintigraphy findings were consistent with sarcoidosis in 96 of 140 patients (69%, 95% CI 61–76%). Whole body ^18^FDG-PET CT, reported in only one study, was performed in 19 of 52 patients and showed abnormalities consistent with sarcoidosis in 15 patients (78%, 95% CI 57–93%).

Serum ACE level was elevated in 238 of 674 patients (35%, 95% CI 32–39%), erythrocyte sedimentation rate was elevated in 64 of 202 patients (32%, 95% CI 25–38%), and hypercalcemia was present in 19 of 222 evaluated patients (9%, 95% CI 5–12%). A Kveim test was performed in eight studies, and was reported positive in 93 of 117 patients (79%, 95% CI 72–87%).

### Treatment

**Fig. 2:**
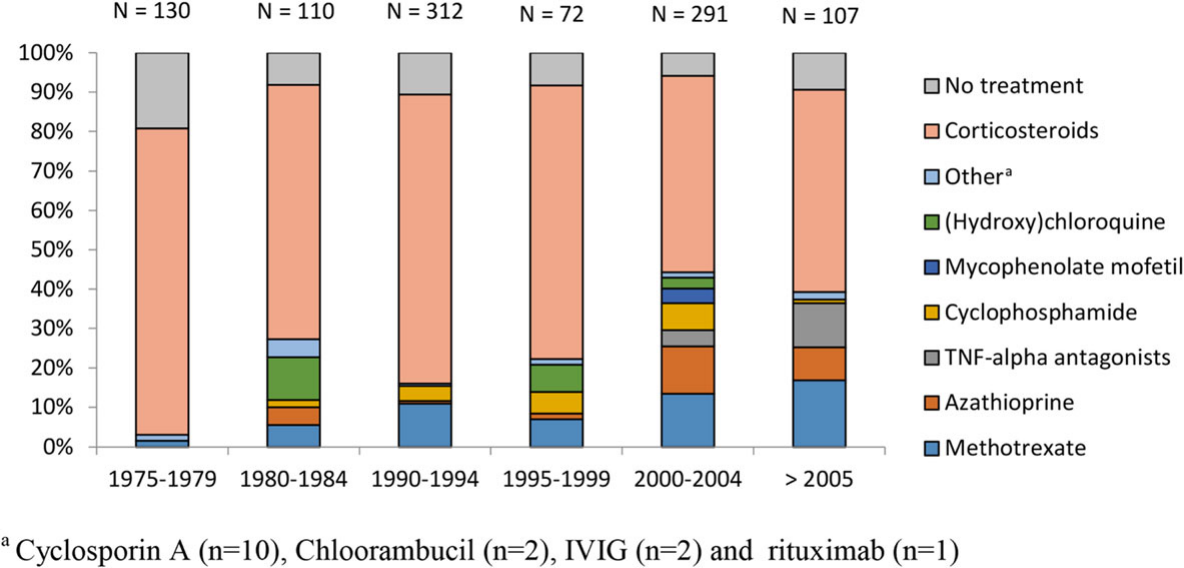
Proportion of treatment used according to timeframe

The treatment strategies are reported in Table 3. No immunosuppressive treatment was indicated in 99 of 655 patients (15%, 95% CI 12–18%). First line therapy, consisting of corticosteroids, was initiated in 434 of 539 patients (81%, 95% CI 77–84%); subsequently, 24% of these patients were switched from first line to second or third line therapy. Second line therapy, consisting of methotrexate, azathioprine, (hydroxyl) chloroquine, mycophenolate mofetil, and cyclosporine A, was initiated in 144 of 539 patients (27%, 95% CI 23–31%). Third line therapy, consisting of cyclophosphamide and TNF-alpha antagonists, was initiated in 49 of 539 patients (9%, 95% CI 7–12%). Overall, 546 of 655 patients (83%, 95% CI 80–86%) were treated with corticosteroids, either as monotherapy or combination therapy with a second or third line agent; methotrexate was administered in 16% (CI 13–19%), azathioprine in 8% (95% CI 6–10%), cyclophosphamide in 6% (95% CI 4–8%), and TNF-alpha antagonists in 4% (95% CI 2–5%). The use of TNF-alpha antagonists increased over time (Fig. 2). Other reported treatments consisted of neurosurgical intervention in 30 of 230 patients (13%, 95% CI 9–17%), hormonal substitution therapy in 18 of 227 patients (8%, 95% CI 4–12%), and anti-epileptic medication in 14 of 99 patients (14%, 95% CI 7–21%).

**Table 3:** Treatment and outcome

Characteristic	n/N^a^ (%)	Characteristic	n/N^a^ (%)
No treatment	99/655 (15)	Other treatment modalities	
First line therapy	434/539 (81)	Neurosurgical intervention	30/230 (13)
Second line therapy	144/539 (27)	Anti-epileptic medication	14/106 (13)
Third line therapy	49/539 (9)	Hormonal substitution	18/227 (8)
Overall treatment		Follow-up, yr., mean (SD)	4.4 (SD 3.15)
Corticosteroids	546/655 (83)	Outcome	
Methotrexate	105/655 (16)	Remission	126/415 (27)
Azathioprine	54/655 (8)	Improvement	147/415 (32)
(Hydroxy) chloroquine	24/655 (4)	Stable disease	100/415 (22)
Mycophenolate mofetil	12/655 (2)	Deterioration	19/415 (4)
Cyclosporine A	10/655 (2)	Mortality	42/826 (5)
Cyclophosphamide	40/655 (6)		
TNF-alpha antagonists	24/655 (4)	Favourable outcome per treatment group	
Treatment switches		First line therapy	161/227 (71)
First to second or third line	88/363 (24)	Second line therapy	47/85 (55)
Second to third line	14/104 (13)	Third line therapy	7/18 (39)
Between third line	7/23 (30)		

^a^ n/N: number for which a certain characteristic is present out of the total number of patients for which it was described

### Outcome

**Fig. 3:**
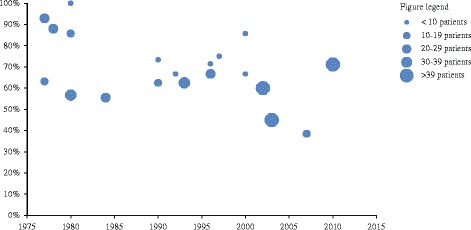
Temporal trend of patients with neurosarcoidosis with reported substantial improvement

Treatment response is reported in Table 3. The average time of follow-up was 4 years (IQR 2–7, SD 4.4). Mortality was reported in 23 of 29 studies (79%), ranging from 0 to 33%. Overall, 42 of 826 patients died (5%; 95% CI 4–7%). The temporal trend of patients with neurosarcoidosis with reported substantial improvement is noted in Fig. 3. Total remission was achieved in 126 of 465 patients (27%, 95% CI 23–31%), incomplete remission in 147 of 465 (32%, 95% CI 27–36%), stable disease in 111 of 465 (24%; 95% CI 20–28%), and deterioration in 28 of 465 patients (6%; 95% CI 4–8%). Favourable outcome was reported in 161 out of 227 patients (71%; 95% CI 65–77%) who received only first line treatment. Favourable outcome was reported in 47 of 85 patients (55%; 95% CI 45–66%) who received second line therapy and were not switched to third line therapy. Seven out of 18 patients (39%; 95% CI 16–62%) who received third line therapy had a favourable outcome.

## Discussion

Our data show that neurosarcoidosis is a diverse illness, with heterogeneous clinical presentation, varying results of ancillary investigation, and considerable inter-individual differences in treatment response. Four clinical presentations could be distilled from our meta-analysis: those presenting with cranial neuropathy, spinal cord inflammation, peripheral neuropathy or myopathy, or (chronic) meningitis. Neurological symptoms were the first clinical manifestation of sarcoidosis in about half of patients, and of those known with sarcoidosis about 5% eventually develop neurosarcoidosis. Although new treatments, such as TNF-alpha antagonists, are increasingly used, the mortality rate among patients with neurosarcoidosis remains 5% and about one third does not have a substantial clinical improvement on treatment.

Our systematic review and meta-analysis has limitations. First, all but one study were retrospective, introducing selection bias. This limits the external validity of our results and may overestimate effect of treatment in this disease. Second, most studies were performed in tertiary referral centres in European countries or the USA, which is also reflected in the ethnicity of included patients, again introducing selection bias. Tertiary care patients might be more severely affected and perhaps less responsive to treatment. Third, studies used heterogeneous inclusion criteria and not all items were reported for all patients. Fourth, patients were included over a period of 30 years. Within this time period, alternative diagnoses mimicking neurosarcoidosis have been described which previously would be classified as probable or even definite neurosarcoidosis. Examples of such mimics are IgG4-related disease or the POEMS syndrome [37–39]. These rare diseases may present with similar clinical features and histopathology. It may well be that different aetiologies may exist for a disease that we now call neurosarcoidosis. Finally, when performing the meta-analyses we found there frequently was significant heterogeneity between studies on the reported data. This confirms the influence of the abovementioned biases.

Making a diagnosis of neurosarcoidosis can be difficult. A definite diagnosis was made only in one out of four patients. The far majority of patients were classified as probable neurosarcoidosis. Diagnostic classification for neurosarcoidosis includes MRI, CSF and serum inflammatory markers, FDG-PET, gallium scan, and chest imaging [10]. Our study shows that none of these diagnostic markers seems to have a good accuracy for the diagnosis. Multicentre prospective diagnostic studies in patients with suspected neurosarcoidosis are needed to test diagnostic accuracy of these tests in the diagnostic process. These studies should determine the value of markers noted in the diagnostic criteria as well as other promising new markers, such as soluble interleukin-2-receptor [35, 40].

Clear guidance for the treatment of patients with neurosarcoidosis is lacking. Most treatment strategies are extrapolated from few studies on pulmonary sarcoidosis. In our meta-analysis 83% of patients received corticosteroids, which can be considered the mainstay of treatment in neurosarcoidosis. However, 24% of the patients initially treated with first line therapy were switched to second or third line therapy. In addition, corticosteroid-associated side effects occur frequently and can be severe [8, 20]. Over time, second and third line therapy emerged into the treatment of neurosarcoidosis, most recently TNF-alpha antagonists. Value of TNF-alpha antagonists has been suggested only in a phase II randomised controlled trial (RCT) in pulmonary sarcoidosis [41]. This trial including 138 patients showed that infliximab therapy resulted in a statistically significant improvement in percentage of predicted forced vital capacity at week 24. The value of TNF-alpha antagonists is based on sporadic case series, and it is unclear if patients benefit from a combined therapy of TNF-alpha antagonists and first and second line therapy. It is also unclear for how long TNF-alpha antagonists should be continued and whether this therapy is associated with side effects. RCTs on treatments or treatment strategies in this disease are urgently needed.

## Conclusions

Neurosarcoidosis develops in 5% of the sarcoidosis patients, and in more than half of these patients neurological symptoms are the primary presentation of sarcoidosis. The diagnosis is difficult due to the heterogeneous clinical presentation and low sensitivity of ancillary investigations. Despite the increasing use of second and third line medication, still one-third of patients do not improve or deteriorate. These data stress the need for prospective cohort studies and treatment trials.

## Additional file

Additional file 1: Table S1. Study descriptives. This includes a summary of all studies included concerning the first author, date of publication, country of study, study design, whether it is a single or multi center study, number of included patients and inclusion period. **Table S2 A-C.** Heterogeneity of variables between studies. This table shows the heterogeneity between studies concerning the various variables. This includes the Q statistic and I^2^. **Table S3.** List of scored variables per study. This is a comprehensive list of all variables we scored per article. (DOC 155 kb)

## Abbreviations

95% CI: 95% confidence interval
ACE: Angiotensin-converting enzyme
CSF: Cerebrospinal fluid
CT: Computer tomography
FDG-PET: Fludeoxyglucose-positron emission tomography
IQR: Interquartile range
MRI: Magnetic resonance imaging
RCT: Randomised controlled trial
SD: Standard deviation
TNF-alpha: Tumor necrosis factor-alpha
