# Supplementary material for: Clinical features, treatment and outcome in neurosarcoidosis: systematic review and meta-analysis
Source: BMC Neurol. 2016 Nov 15;16:220. doi: 10.1186/s12883-016-0741-x (PMC5109654; doi:10.1186/s12883-016-0741-x)
Supplement: Additional file 1: Table S1. — Study descriptives. This includes a summary of all studies included concerning the first author, date of publication, country of study, study design, whether it is a single or multi center study, number of included patients and inclusion period. Table S2 A-C. Heterogeneity of variables between studies. This table shows the heterogeneity between studies concerning the various variables. This includes the Q statistic and I2. Table S3. List of scored variables per study. This is a comprehensive list of all variables we scored per article. (DOC 155 kb) [file 12883_2016_741_MOESM1_ESM.doc]

**Supplementary table 1.** Study descriptives

| **Ref.** | **First author** | **Date of publication** | **Country** | **Study design** | **Single/multicentre** | **Patients**  **patients** | **Inclusion period** |
| --- | --- | --- | --- | --- | --- | --- | --- |
| 6 | Allen | 2003 | Australia | Prospective | Single | 32 | 1991 - 1994 |
| 8 | Zajicek | 1999 | United Kingdom | Retrospective | Multi | 68 | 1970 - 1995 |
| 9 | Marangoni | 2006 | Italy | Retrospective | Single | 7 | 1989 - 2003 |
| 11 | Elkin | 1985 | South Africa | Retrospective | Single | 5 | 1977 - 1982 |
| 12 | Pentland | 1985 | United Kingdom | Retrospective | Single | 19 | 1970 - 1983 |
| 13 | Stern | 1985 | USA | Retrospective | Single | 33 | 1975 - 1980 |
| 14 | Oksanen | 1986 | Finland | Retrospective | Single | 50 | 1969 - 1983 |
| 15 | Chen | 1989 | Australia | Retrospective | Single | 14 | 1973 - 1987 |
| 16 | Chapelon | 1990 | France | Retrospective | Single | 35 | 1965 - 1988 |
| 17 | Zouaoui | 1992 | France | Retrospective | Single | 9 | Unknown |
| 18 | Radziwill | 1995 | Switzerland | Retrospective | Single | 8 | 1985 - 1995 |
| 19 | Lower | 1997 | USA | Retrospective | Single | 71 | 1986 - 1995 |
| 20 | Sharma | 1997 | USA | Retrospective | Single | 37 | 1965 - 1995 |
| 21 | Briner | 1998 | Switzerland | Retrospective | Single | 6 | 1987-1997 |
| 22 | Pavese | 1999 | France | Retrospective | Single | 16 | 1982 - 1998 |
| 23 | Ferriby | 2001 | France | Retrospective | Multi | 27 | 1968 - 1999 |
| 24 | Heuser | 2004 | Norway | Retrospective | Single | 15 | 1990 - 2001 |
| 25 | Kellinghaus | 2004 | Germany | Retrospective | Single | 13 | 1993 - 2000 |
| 26 | Karouache | 2004 | Morocco | Retrospective | Single | 9 | 1995 - 2004 |
| 27 | Spencer | 2004 | USA | Retrospective | Single | 21 | 1988 - 2003 |
| 28 | Joseph | 2008 | United Kingdom | Retrospective | Single | 30 | 1990 - 2012 |
| 29 | Pawate | 2009 | USA | Retrospective | Single | 54 | 1995 - 2008 |
| 30 | Shah | 2008 | USA | Retrospective | Multi | 32 | 1999-2008 |
| 31 | Gascón-Bayarri | 2011 | Spain | Retrospective | Single | 30 | 1974 - 2005 |
| 32 | Nozaki | 2012 | USA | Retrospective | Single | 70 | 1995 - 2010 and 1997 - 2011 |
| 33 | Carlson | 2014 | USA | Retrospective | Multi | 305 | 2000-2013 |
| 34 | Gözübatik-Celik | 2015 | Turkey | Retrospective | Single | 7 | 2006 - 2013 |
| 35 | Wegener | 2015 | Switzerland | Retrospective | Multi | 13 | 2000-2014 |
| 36 | Leonard | 2015 | Netherlands | Retrospective | Single | 52 | 1996-2015 |

**Supplementary table 2A.** Heterogeneity of variables between studies (patient characteristics)

| **Characteristic** | **Q statistic/I^2^** | **Characteristic** | **Q statistic/I^2^** |
| --- | --- | --- | --- |
| Sex | 38/26 | Neurological symptoms  eeasdfexxaexamination |  |
| Ethnicity |  | Headache | 63/65 |
| Caucasian | 108/87 | Sensory abnormalities | 68/73 |
| African | 150/90 | Hypaesthesia | 10/32 |
| Presentation of disease |  | Paraesthesia | 35/66 |
| History of sarcoidosis | 28/35 | Neuropathic pain | 18/66 |
| Systemic sarcoidosis at admission | 54/67 | Gait abnormalities | 17/48 |
| Primary neurological presentation | 98/76 | Visual impairment | 36/55 |
| Isolated neurosarcoidosis | 70/79 | Fatigue | 15/46 |
| Systemic involvement |  | Motor abnormalities | 62/66 |
| Pulmonary | 76/74 | Hemiparesis | 28/42 |
| Eye | 38/47 | Paraparesis | 25/48 |
| Dermatological | 24/17 | Ataxia | 31/51 |
| Rheumatological | 46/57 | Vertigo | 19/52 |
| Otorhinolaryngeal | 32/38 | Hearing impairment | 25/40 |
| Hepatic | 24/16 | Seizures | 29/15 |
| Cardial | 12/0 | Nausea | 4/0 |
| Constitutional symptoms | 81/75 | Diplopia | 18/32 |
| Cranial nerve palsy |  | Micturition abnormalities | 13/18 |
| n. VII palsy | 75/71 | Dysarthria | 7/8 |
| n. II palsy | 70/69 | Dysphagia | 5/0 |
| n. V palsy | 40/43 | Psychiatric symptoms | 13/26 |
| n. VIII palsy | 34/40 | Nystagmus | 10/39 |
| n. VI palsy | 22/0 | Papilledema | 19/43 |
| n. III palsy | 15/0 | Site of neurological involvement | |
| n. IX-X palsy | 13/0 | Spinal cord disease | 40/60 |
| n. I palsy | 19/0 | Peripheral neuropathy | 70/77 |
| n. IV palsy | 21/14 | Polyneuropathy | 43/70 |
| n. XI palsy | 13/0 | (mult.) mononeuropathy | 8/53 |
| n. XII palsy | 13/0 | Radiculopathy | 5/14 |
| > 1 cranial nerve involved | 71/75 | Meningitis | 63/67 |
|  |  | Myopathy | 15/39 |
|  |  | Neuro-endocrine | 38/35 |
|  |  | Hydrocephalus | 31/35 |

**Supplementary table 2B.** Heterogeneity of variables between studies (ancillary investigations on presentation)

| **Characteristic** | **Q statistic/I^2^** | **Characteristic** | **Q statistic/I^2^** |
| --- | --- | --- | --- |
| Blood chemical tests |  | Abnormal ancillary investigation |  |
| Serum ACE increased | 58/65 | Chest X-ray | 65/74 |
| Serum calcium increased | 11/45 | Chest CT | 13/20 |
| ESR >20 mm/hr | 35/80 | Gallium-67 scintigraphy | 14/44 |
|  |  | Cranial CT | 12/0 |
| Cerebral spinal fluid analysis |  | Cranial MRI | 34/42 |
| Lumbar puncture performed | 129/81 | Parenchymal lesions | 39/51 |
| White cell count ( > 5cells/mm^3^) | 37/43 | Meningeal enhancement | 98/85 |
| Protein (> 0.45 g/L) | 32/34 | Mass lesions | 8/0 |
| Hypoglycorrhachia | 24/41 | Cranial nerve enhancement | 18/60 |
| Increased IgG-index | 6/0 | Spinal MRI | 37/68 |
| Oligoclonal bands present | 21/48 | Diagnosis |  |
| Increased CSF ACE | 26/66 | Histopathological confirmation | 72/69 |
| Normal | 23/21 | Definite neurosarcoidosis | 28/64 |
|  |  | Probable neurosarcoidosis | 38/74 |
|  |  | Possible neurosarcoidosis | 4/0 |

**Supplementary table 2C.** Heterogeneity of variables between studies (treatment and outcome)

| **Characteristic** | **Q statistic/I^2^** | **Characteristic** | **Q statistic/I^2^** |
| --- | --- | --- | --- |
| No treatment | 60/60 | Other treatment modalities |  |
| First line therapy | 59/65 | Neurosurgical intervention | 8/2 |
| Second line therapy | 88/76 | Anti-epileptic medication | 3/0 |
| Third line therapy | 58/64 | Hormonal substitution | 17/53 |
| Overall treatment |  |  |  |
| Corticosteroids | 60/60 | Outcome |  |
| Methotrexate | 64/63 | Remission | 36/52 |
| Azathioprine | 53/55 | Improvement | 40/58 |
| (Hydroxy)chloroquine | 49/51 | Stable disease | 33/48 |
| Mycophenolate mofetil | 29/15 | Deterioration | 13/0 |
| Cyclosporine A | 10/0 | Mortality | 28/21 |
| Cyclophosphamide | 38/38 | Favourable outcome per |  |
| TNF-alpha inhibitors | 33/28 | treatment group |  |
| Treatment switches |  | First line therapy | 22/23 |
| First to second or third line | 57/65 | Second line therapy | 9/0 |
| Second to third line | 23/19 | Third line therapy | 4/0 |
| Between third line | 28/21 |  |  |

**Supplementary table 3.** List of scored variables per study

| Study characteristics | Blood chemistry |
| --- | --- |
| First author | ACE elevated |
| Year of publication | Ca elevated |
| Start study period | ESR elevated |
| End study period | Cerebrospinal fluid examination |
| Study duration | Performed |
| Country | Elevated pressure |
| Multicentre/Single centre | Pleiocytosis |
| Prospective/Retrospective | Elevated protein level |
| Follow-up time | Glucose |
| No of included patients | IgG index |
| Inclusion criteria | Oligoclonal bands |
| Age (mean/median) | ACE elevated |
| Gender | Soluble IL-2 receptor elevated |
| Ethnicity | Normal CSF |
| Zajicek criteria | Imaging investigations |
| Disease manifestation | MRI-Brain |
| History of sarcoidosis | MRI-spine |
| Primary neurological presentation | X-thorax |
| Clinically isolated neurosarcoidosis | CT-thorax |
| Systemic involvement at presentation | Gallium scan |
| Systemic involvement after diagnosis neurosarcoidosis | PET-CT |
| Acute, subacute or chronic presentation | Other ancillary investigations |
| Clinical characteristics | BAL |
| Cranial nerve palsy | Kveim test |
| Chronic meningitis | Outcome/treatment response |
| Seizures | Remission |
| Impaired consciousness | Incomplete remission |
| Headache | Stable disease |
| Motor symptoms | Deterioration |
| Movement disorders | Death |
| Sensory symptoms | Relaps |
| Cognitive symptoms | Treatment |
| Psychiatric symptoms | First line therapy |
| Endocrinopathy | Second line therapy |
| Cerebellar symptoms | Third line therapy |
| Brain stem symptoms | Treatment switches |
| Vascular complications | Other treatment modalities^b^ |
| Peripheral neuropathy | Individual immunosuppressive agents^c^ |
| Myopathy |  |
| Myelopathy |  |
| Other symptoms^a^ |  |

^a^ Ataxia, nystagmus, vertigo, diplopia, papilledema, dysarthria, dysphagia, micturition abnormalities, Visual impairment, hearing impairment, myalgia, hydrocephalus, muscle spasm, abnormal gait, fatigue, nausea, vomiting.

^b^ Neurosurgical treatment, endocrine treatment, symptomatic treatment, anti-epileptic medication, radiation therapy.

^c^ Corticosteroids, methotrexate, azathioprine, (hydroxyl)chloroquine, mycophenolate, Cyclosporine A, Chloorambucil, Interferon, IvIg, Cyclophosphamide, TNF-alpha antagonists.
